# Prevalence of common mental disorders in adult Syrian refugees resettled in high income Western countries: a systematic review and meta-analysis

**DOI:** 10.1186/s12888-021-03664-7

**Published:** 2022-01-05

**Authors:** Thomas P. Nguyen, Maria Gabriela Uribe Guajardo, Berhe W. Sahle, Andre M. N. Renzaho, Shameran Slewa-Younan

**Affiliations:** 1 Mental Health, School of Medicine, Western Sydney University, Sydney, Australia; 2 School of Nursing and Midwifery, Deakin University, Geelong, Victoria Australia; 3 Centre for Quality and Patient Safety Research (QPS), Alfred Health Partnership, Melbourne, Victoria Australia; 4 Centre for Mental Health, Melbourne School of Population and Global Health, University of Melbourne, Melbourne, Victoria Australia; 5 Translational Health Research Institute, School of Medicine, Western Sydney University, Sydney, Australia; 6 Maternal, Child and Adolescent Health Program, Burnet Institute, Melbourne, Victoria Australia

**Keywords:** Syrian refugees, Mental illness, Posttraumatic stress disorder, Depression, Generalized anxiety disorder, Prevalence rate, High-income country

## Abstract

**Background:**

The immense social upheaval and ongoing humanitarian crisis created by the 2011 war in Syria has forced millions of civilians to flee their homeland, many of whom seek refugee status in Western nations. Whilst it is known that the prevalence of mental illness is higher within refugee populations, this systematic review and meta-analysis aims to pool the prevalence rates of common mental disorders (namely posttraumatic stress disorder, depression and generalized anxiety disorder) in adult Syrian refugees resettled in high income Western countries.

**Methods:**

Seven electronic databases (Medline, PsychInfo, CINAHL, PTSDpubs, SCOPUS, PubMed and Embase) were searched up to the 31st of December 2020. Using pre-determined inclusion and exclusion criteria, relevant articles were screened by title and abstract, and later by full text. A meta-analysis was used to estimate the prevalence rates for each mental illness.

**Results:**

Eleven studies met the eligibility criteria for the systematic review. Nine of these studies had a low-moderate risk of bias and were included in the meta-analysis. Of the 4873 refugees included in the meta-analysis, the total pooled prevalence rate of having any of the three mental disorders was 33% (CI 95%, 27-40%), 40% for anxiety (CI 95%, 31-50%), 31% for depression (CI 95%, 20-44%) and 31% for PTSD (CI 95%, 22-41%). A meta-regression revealed that the total pooled prevalence rate for having any of the three mental disorders was not influenced by age, host country, duration in host country, educational or marital status.

**Conclusions:**

Despite significant study heterogeneity, the prevalence rates of common mental disorders in adult Syrian refugees resettled in high-income Western countries are significantly higher than reported rates in the general population.

**Supplementary Information:**

The online version contains supplementary material available at 10.1186/s12888-021-03664-7.

## Introduction

In 2020, the United Nations High Commissioner for Refugees (UNHCR) reported that the number of forcibly displaced people had reached a record high of 82.4 million people [1]. Of this growing population, it is estimated that 26.4 million people are of refugee status – the recognition that one has been forced to flee their country due to war, violence or the well-founded fear of persecution [1, 2]. The UNHCR reports that the current global refugee population has almost doubled since 2012, owing greatly to the outbreak of the war in Syria beginning in March 2011 [3]. As of March 2021, the war in Syria has been responsible for over 350,000 deaths and the upheaval of 5.6 million Syrian civilians [4, 5].

Early on in the conflict, many Syrian civilians moved into makeshift settlements in neighbouring Turkey, Lebanon and Jordan [6]. The prolonging of what was initially viewed as a temporary conflict meant that these nations were unable to keep up with the influx of displaced people [6]. These unstable living arrangements were partly responsible for the movement of over one million Syrian refugees into Europe in 2015 alone [6]. In response to this global crisis, government and UNHCR-facilitated humanitarian programs in Western countries have committed to hosting a sizeable proportion of the Syrian refugee population [3]. For example, Germany has resettled over half a million Syrian refugees whilst Sweden has resettled over 100,000 [3]. However, the majority of Syrian refugees remain in neighbouring Turkey, Lebanon and Jordan where employment and civil rights are limited and living conditions are poor [7].

Regardless of their current migration and resettlement status, it is well established that many refugees will have been exposed to potentially traumatic events (PTEs) at some point before or throughout their migratory trajectory [8]. PTEs may arise following exposure to war, violence or torture, with most refugees reporting experiences such as the death of loved ones, prolonged deprivation and extended separation from family members during their migration [8, 9]. Exposure to PTEs are known to adversely affect psychological wellbeing, manifesting in common mental disorders such as posttraumatic stress disorder (PTSD) and depression [8, 9].

The experience of post-migration stressors in a resettled refugee population may also exacerbate or precipitate a mental illness. These may include struggling to negotiate changes to one’s personal and national identity, prolonged separation from family and friends or the acculturative stress associated with resettling in a foreign country which may present significant changes in culture, language and environment from their country of origin [10, 11]. Past research on refugees resettled in high-income Western countries have demonstrated female gender, higher levels of education, greater exposure to traumatic events, less time spent in the host nation and post-migration stressors (e.g., unemployment, poverty and discrimination) have been associated with a greater risk of PTSD and depression [8, 10, 12]. However, consensus on what age groups are most vulnerable to mental illness in the refugee population remains contentious [10, 13].

Henkelmann and colleagues (2020) conducted a systematic review and meta-analysis of the prevalence rates of mental illness in refugees resettling in high-income countries. From 66 studies, they reported a pooled prevalence rate of 29 and 37% for diagnosed and self-reported PTSD, 30 and 40% for diagnosed and self-reported depression, with 13 and 42% for diagnosed and self-reported anxiety [12]. They also found that the pooled prevalence rates were not predicted by mean age, gender, average duration of residence or methodological quality [12]. However, their review did not account for the heterogeneity exhibited by the varying countries of origin.

To provide the appropriate mental health care for the large number of Syrian refugees that have sought asylum and resettled in Western nations, a clear understanding of the prevalence rates of common mental disorders is required. This knowledge forms the basis of culturally sensitive care which is paramount in a population that also shares different values, help-seeking behaviours and beliefs about the causes of mental illness [14]. With clinicians based in Western countries increasingly seeing Syrian refugees in their practices due to the ongoing humanitarian crisis, culturally sensitive care is crucial in ensuring that mental illness in this vulnerable population is detected early and treated for in a culturally appropriate way.

Peconga et al.’s (2019) recent review goes in some ways to estimating the weighted average prevalence rates of PTSD (43.0%), depression (40.9%) and anxiety (26.6%) in adult Syrian refugees [15]. However, limitations such as the lack of study quality appraisals and risk of bias assessments, the inclusion of multiple studies that utilized the same samples in addition to combining study populations residing in low and or middle income countries (LMIC) with high-income Western countries confounds their findings [15–18]. Given that eight out of the 15 studies included used study populations exclusively from LMICs, the authors’ conclusions regarding Syrian refugees as a homogenous group may have been skewed, given the differences in migration and resettlement status between asylum seekers and refugees in LMIC with refugees resettled in high-income Western countries [15]. Furthermore, given asylum seekers are significantly more likely to report symptoms of PTSD, depression and anxiety, the inclusion of asylum seekers does not accurately represent the experiences of refugees resettled in high-income Western countries [8, 19]. These limitations are important considerations given it is well established that PTEs are heterogenous in nature and may impact individuals in different ways due to cultural and socio-economic nuances [20].

Thus, this systematic review aimed to overcome these existing limitations by synthesising the available studies that report on the prevalence rates of common mental disorders in Syrian adult refugees resettled in high-income Western nations. Moreover, by undertaking a meta-analysis, the prevalence rates of common mental disorders (namely PTSD, depression and generalized anxiety disorder) were estimated while accounting for methodological quality and risk of bias in the studies included. A meta-regression was also conducted to assess whether the prevalence rates reported are influenced by age, host country, duration in host country, educational status or marital status.

## Methods

In accordance with Preferred Items for Reporting of Systematic Reviews and Meta Analyses (PRISMA) guidelines and the updated 2020 PRISMA statement (see Additional file 1), we conducted an electronic search of all English-language literature from seven databases, from January 1st, 2011 to December 31st, 2020 [21]. The protocol for this review was registered on the Open Science Framework in December 2020: https://osf.io/e46cz/.

### Eligibility criteria

Studies that measured the prevalence rates of at least one common mental disorder (PTSD, depression and generalized anxiety disorder) in Syrian adult refugees (> 18 years) who have resettled in a high income Western country were considered eligible for this review. We limited our definition of high-income Western countries to nations in Europe, North America and Australasia that partake in the UNHCR’s resettlement programme to ensure definitional consistency (i.e., all eligible studies had formal, government-approved programs to grant refugee status to individuals originating from Syria) [22]. Only those studies that utilised validated quantitative screening tools or formal diagnoses of a common mental disorders, in line with either the criteria set by the International Classification of Diseases (ICD), Diagnostic and Statistical Manual of Mental Disorders (DSM), Fourth Edition or DSM Fifth Edition were included. Studies that included samples with only Syrian asylum seekers, internally displaced persons (IDPs) or refugees resettled in non-Western countries were excluded. We excluded samples with asylum seekers and IDPs as their legal status and protections are different and have been found in a prior meta-analysis to report significantly higher prevalence rates of mental illness than refugees [8].

We considered all study designs for this review except for systematic reviews, meta-analyses, editorials and articles with qualitative-only methods. Other exclusion criteria included studies not published in English, studies published prior to 2011, clinical samples, studies that repeated samples from prior studies and publications that were not peer-reviewed.

### Search strategy

Systematic searches were conducted on the 28th of December 2019 and 1st of August 2020 by one of the authors (TPN) on the following six electronic databases: Medline (Ovid), PsycInfo (Ovid), CINAHL, PTSDpubs, SCOPUS, PubMed. Embase was additionally searched on the 23rd of December 2020 and all electronic databases were searched again on the 25th of January 2021 for articles up and until the 31st of December 2020. This was completed using keyword searches, free search terms and their associated MeSH headings. MeSH headings used included ‘Refugees’, ‘Syria’ and ‘Mental Disorders’. These searches were replicated as closely as possible across the six databases (see Additional file 1 for search strings used).

Terms used for the search in PubMed are outlined below:

((refugee* or asylum seeker* or migrant* or immigrant* or displaced person*) AND (Syria* or (Syria* ADJ (Assyrian or Arabs or Jews or Kurds or Orthodox or Shia or Turkmen or Turkoman or Circassian or Alawis or Isma'ilis or Druzes or Armenian))) AND (mental adj (illness* or disorder* or health or problem) or psychiatric adj (illness* or disorder* or symptom* or comorbidity*) or PTSD or post-traumatic stress disorder or posttraumatic stress disorder or post-traumatic stress disorder or posttraumatic or trauma* or depress* or anxi*))

Following the identification of these articles and the exclusion of all duplicate records, two reviewers (TPN and GU) independently screened all articles based on their title and abstract for inclusion based on the eligibility criteria described above. Any discrepancies between the two reviewers were revisited again by both reviewers, with a third reviewer (SSY) brought in to reach a consensus if there were still any disagreements. All articles that met our eligibility criteria were reviewed based on their full text by two reviewers (TPN and GU). Discrepancies were also discussed again by both reviewers, with a third reviewer (SSY) brought in to reach a consensus if there were still any disagreements.

### Data extraction

Two reviewers (TPN, GU) worked independently to extract data from the included studies utilising a standardised and agreed upon form. One reviewer checked both forms for any inconsistencies and consulted with the other reviewer until a consensus was reached. Data extracted included study design and location, sampling method, prevalence rates of common mental disorders (PTSD, depression and anxiety) as well as the measurement tool used. We also extracted information on a small number demographic datapoints which have been previously reported in the literature as potential variables that may influence the prevalence rates of common mental disorders in resettled refugees (i.e., number of males, females, age range and mean, mean duration in host nation, marital status, education attainment levels and exposure to potentially traumatic events.) Further information was sought from the corresponding authors if it was not provided in the published article.

### Methodological quality and risk of Bias

Methodological quality and risk of bias was independently assessed by two reviewers (TPN, GU) using the JBI Critical Appraisal Checklist for Prevalence Studies. The JBI tool uses nine criteria to assess the methodological quality and determine how well a study has addressed potential biases in its design and analysis [23]. Studies scoring five or more out of nine were included in the meta-analysis as they were deemed to have a low to moderate risk of bias. Discrepancies were discussed by both reviewers, with a third reviewer (SSY) brought in to reach a consensus if there were still any disagreements.

### Statistical analysis

A summary table was prepared to ensure that all relevant data, including demographic characteristics, study design, sample size, and outcome assessment tools were extracted in a consistent way. The ‘*metaprop’* function in Stata version 16.0 was used to produce forest plots of the overall and sex-specific pooled prevalence of all three mental disorders and their associated 95% confidence intervals (CIs) [24]. Meta-analysis was performed using the Freeman-Tukey double arcsine transformed proportions to account for variance instability. The random-effects model was preferred due to significant heterogeneity among the included studies (I^2^ > 50%). Subgroup analyses were performed to compare the prevalence of all three mental disorders by gender. We used a meta-regression to investigate whether the prevalence of mental disorders varied by demographic characteristics (i.e. age, gender, duration in host country, educational or marital status.) We assessed heterogeneity between meta-analyses using the I^2^ statistic [25]. Each meta-analysis was judged to have either low (25%), moderate (50%) or high (75%) heterogeneity, based off established cut-off points [25]. Publication bias was assessed using Begg’s adjusted rank correlation test, Egger’s regression asymmetry test, and visual inspection of funnel plots [26].

## Results

**Fig. 1:**
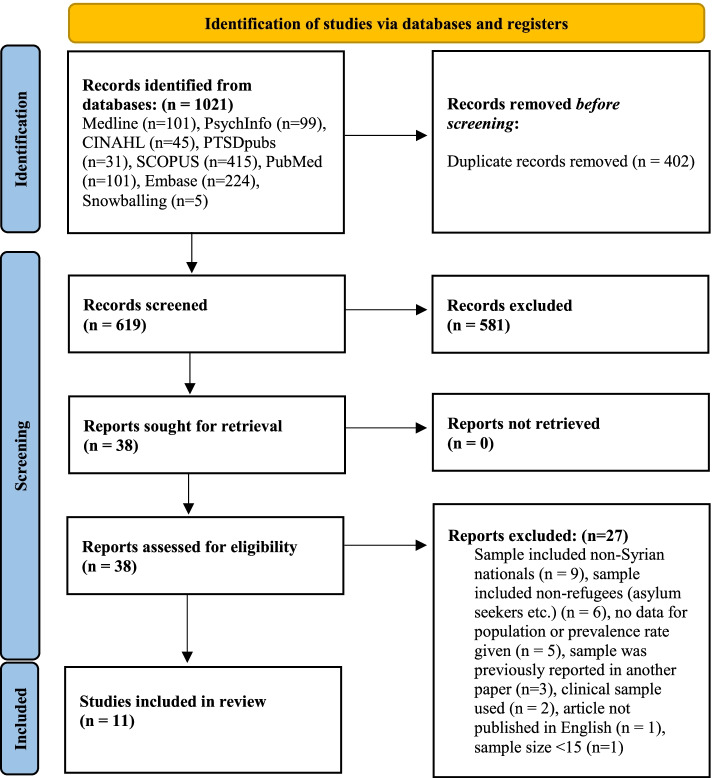
Flowchart search strategy

Figure 1 provides a summary of the flowchart search strategy from the systematic review. Of the seven databases searched up and until the 31st of December 2020, our searches yielded 619 unique studies. Based on title and abstract screening, 38 studies were eligible for full text review, with 11 of these studies being included in the final systematic review. One study met all eligibility criteria but was excluded because the publication of the full-text article was not in English [27].

Table 1 provides a demographic summary of all the included articles from the systematic review. Of the 11 studies, seven papers reported on refugees resettling in Europe (Germany *n* = 3, Sweden *n* = 2, Netherlands *n* = 1, Norway n = 1), three in North America (USA n = 2, Canada n = 1) and one from Australia [18, 28–37]. Two studies were of longitudinal design, nine were cross-sectional with one including mixed-methods methodologies [18, 28–37]. Most studies (*n* = 9) used either convenience, snowballing or chain referral sampling whilst two studies used registry, population-based sampling methods [18, 28–37].

**Table 1:** Demographics of included studies

Authors and year	Study design	Study Location	Sampling method	Outcome	Study setting	Mean time in host nation	Education Level	Marital status	Employment	No. of traumatic events	Age mean (SD)
Ahmad et al., 2020 [27]	Longitudinal	Canada	Snowballing, public announcements	Predictors of depression-level symptoms at baseline and one-year post-resettlement	Community	M = 13.4 months (SD = 5.5 months)	28.8% (*n* = 554) university level or higher, 44.7% (*n* = 860) secondary/high school, 26.5% (*n* = 509) none/primary school	22.1% (*n* = 425) single, 72.2% (*n* = 1384) married, 5.7% (*n* = 109) separated/divorced/widowed	23.3% (*n* = 448) employed, 76.7% (*n* = 1476) unemployed	N/A	38.5 (13.8)
Al Ibraheem et al., 2017 [28]	Cross-sectional	Netherlands	Snowballing or chain referral sampling	Effects of trauma on the physical and mental health of Syrian refugees	Community	N/A	N/A	N/A	N/A	M = 6.5, SD = 3.2	32.9 (6.9)
Chung et al., 2018 [19]	Cross-sectional	Sweden	Convenience	Relationship between trauma exposure, trauma centrality, emotional suppression, and psychiatric comorbidity	Community	M = 15.5 months (SD = 12.2 months)	60.3% (*n* = 340) university, 39.2% (*n* = 221) secondary school, *n* = 3 did not attend school	53.2% (*n* = 300) married, 43.1% (*n* = 243) single, 3.7% (*n* = 21) divorced/separated	100% (*n* = 564) unemployed	M = 8.4, SD = 4.5	35.3 (11.8)
Euteneuer et al., 2018 [29]	Cross-sectional	Germany	Convenience	Subjective social mobility and depressive symptoms	Community	M = 11.4 months (SD = 7.0 months)	M = 13.6 years, SD = 2.7	N/A	N/A	N/A	28.8 (8.2)
Georgiadou et al., 2018 [30]	Cross-sectional	Erlangen, Germany	Registry-based	Mental distress	Community	M = 23.3 months (SD = 6.5 months)	M = 10.2 years, SD = 4.5; 6% (*n* = 12) no education, 24.5% (*n* = 49) primary education, 42% (*n* = 84) secondary education, 22% (n = 44) tertiary education	59.5% (*n* = 119) married, 37% (*n* = 74) single, 3.5% (*n* = 7) divorced/widowed	100% (*n* = 518) unemployed	M = 2.3, SD = 2.4	33.3 (10.6)
Javanbakht et al., 2019 [31]	Cross-sectional	USA	Recruited at mandatory 1 month health screening	Brief mental health screening for three common psychiatric consequences of exposure to stress and trauma	Community	N/A	7.8% (n = 12) college, 52.6% (*n* = 81) high school, 32.5% (n = 50) elementary/middle school, 7.1% (n = 11) illiterate	74.4% (*n* = 116) married, 20.5% (*n* = 32) single, 1.9% (*n* = 3) divorced, 3.2% (n = 5) widowed	100% (*n* = 157) unemployed	N/A	36.1 (11.4)
Lies et al., 2020 [32]	Longitudinal	Australia	Snowballing	Post-migration stress, mental health (PTSD, anxiety, depression), and sleep symptoms	Community	M = 29.7 months (SD = 6.4 months)	25% (*n* = 17) primary school; 47.5% (*n* = 33) secondary education; 27.5% (n = 19) tertiary education	66.7% (*n* = 46) married; 24.6% (n = 17) single; 8.7% (*n* = 6) widowed	5.8% (n = 4) employed, 94.2% (*n* = 65) unemployed	M = 16.9, SD = 5.6	45.6 (15.9)
M’zah et al., 2019 [33]	Cross-sectional	Atlanta, USA	Convenience	Post migration stressors and mental health condition symptoms	Community	M = 11 months	36% (n = 9) secondary education, 28% (n = 7) tertiary education	92% (*n* = 23) married, 4% (n = 1) single, 4% (*n* = 1) widowed)	48% (n = 12) employed, 52% (n = 13) unemployed	M = 5.8	37.5 (9.4)
Strømme et al., 2020 [34]	Cross-sectional	Norway	Convenience	Health status and associations between migration related exposures and both chronic pain and mental health	Community	M = 1.5 years (SD = 1 year)	M = 11.2 years, SD = 4 years	44% (n = 138) single, 50% (*n* = 156) married, 5% (*n* = 16) separated/divorced/widowed, 2% (*n* = 5) other	N/A	40% sample experienced a traumatic event	31.2 (8.9)
Tinghög et al., 2017 [35]	Cross-sectional, population-based	Sweden	Randomized from registry	Mental ill health comorbidity	Community	All > 3 years ≤2011 (6.5%) 2012 (27.5%) 2013 (66.0%)	0–9 years (40.2%), > 9 years without a university degree (21.0%), > 12 years with a university degree (38.7%)	63.5% married, 31.8% unmarried, 4.8% divorced or widowed	N/A	M = 4.2	N/A
von Haumeder et al., 2019 [36]	Mixed-methods, cross-sectional	Germany	Convenience	Trauma-related coping self-efficacy, resilience, and environmental factors as predictors of psychological adaptation and PTSD	Community	M = 23.7 months	15.7% (*n* = 20), ≤ 8th grade, 17.3% (n = 22) 9th-11th grade, 15.0% (n = 19) high school graduate, 22% (*n* = 28) some college, 29.9% (*n* = 38) 4 years of college or more	43.3% (n = 55) married and living together, 56.7% (*n* = 72), single/divorced/widowed/married but not living together	15.7% (n = 20) employed, 16.5% (n = 21) homemaker, 33.1% (n = 42) student, 33.9% (*n* = 43) unemployed/disabled/retired	N/A	31.9 (10.7)

*N/A* not available, *M* mean, *SD* standard deviation

Table 2 summarizes the sample sizes, mental health outcomes and assessment tools used by each study. Sample sizes ranged from *n* = 25 to *n* = 1924 [28, 34]. One study exclusively looked at male Syrian refugees whilst two studies did not report the prevalence rates by gender [29, 32, 34]. Nine studies assessed for PTSD, seven looked at depression and five measured anxiety [18, 28–37]. Only one study used a diagnostic interviewing assessment tool to measure mental health outcomes whilst the other ten studies used screening assessment tools [18, 28–37]. Seven different tools were used to measure PTSD (CAPS-2, HTQ, ETI, PCL-C, PTSD-9, PCL-5 and PTSD-8), with the HTQ (*n* = 3) being the most common amongst the nine studies assessing PTSD [18, 28–37]. From the seven studies looking at depression, four used the HSCL-25 and three used the PHQ-9 [28, 31–34, 36, 37]. Four studies also used the HSCL-25 to assess for anxiety whilst the one paper used the GAD-7 [31, 33, 34, 36, 37].

**Table 2:** Summary of mental health outcomes and assessment tools used

Author and Year	Mental illness	Tool used	Females			Males			Total		
			Sample size	Prevalence rate (%)	No. of cases	Sample size	Prevalence rate (%)	No. of cases	Sample size	Prevalence rate (%)	No. of cases
**Ahmad et al. 2020** [27]	Depression	Patient Health Questionaire (PHQ)-9	984	18.5	182	937	11.6	109	1924	15.2	293
**Al Ibraheem et al. 2017** [28]	PTSD	Clinician-Administered PTSD Scale (CAPS-2)	N/A	N/A	N/A	N/A	N/A	N/A	111	23.4	26
**Chung et al. 2018** [19]	PTSD	Harvard Trauma Questionnaire	183	31.7	58	381	28.9	110	564	30	168
**Euteneuer et al. 2018** [29]	Depression	Patient Health Questionaire (PHQ)-9 (Arabic version)	–	–	–	164	28.7	47	164	28.7	47
**Georgiadou et al. 2018** [30]	PTSD	Essen Trauma Inventory (ETI)	61	16.4	10	139	8.6	12	200	11.4	22
	Depression	Patient Health Questionnaire—Depression Module (PHQ-9)	61	34.4	21	139	23.7	33	200	27	54
	Anxiety	GAD-7 Scale	61	24.6	15	139	8.6	12	200	13.5	27
**Javanbakht et al. 2019** [31]	PTSD	PTSD Checklist Civilian version (PCL-C) DSM-IV version	74	31.9	22	83	32.5	25	157	32.2	47
	Depression	Hopkins Symptom Checklist 25 items (HSCL-25)	74	58.8	30	83	38.3	23	157	47.7	53
	Anxiety	Hopkins Symptom Checklist 25 items (HSCL-25)	74	52.7	29	83	29.7	19	157	40.3	48
**Lies et al. 2020** [32]	PTSD	Short posttraumatic stress disorder inventory (PTSD-8)	31	77.4	24	38	50	19	69	62.3	43
	Depression	Hopkins Symptom Checklist 25 items (HSCL-25)	31	68	21	38	42.1	16	69	53.6	37
	Anxiety	Hopkins Symptom Checklist 25 items (HSCL-25)	31	74	23	38	36.8	14	69	53.6	37
**M’zah et al. 2019** [33]	PTSD	PTSD Checklist for DSM-5 (PTSD-9)	10	N/A	N/A	15	N/A	N/A	25	84	21
	Depression	Hopkins Symptom Checklist 25 items (HSCL-25)	10	N/A	N/A	15	N/A	N/A	25	44	11
	Anxiety	Hopkins Symptom Checklist 25 items (HSCL-25)	10	N/A	N/A	15	N/A	N/A	25	60	15
**Strømme et al. 2020** [34]	PTSD	Harvard Trauma Questionnaire (HTQ)	73	4	3	198	11	21	271	9	24
**Tinghög et al. 2017**^a^ [35]	PTSD	Harvard Trauma Questionnaire (HTQ)	452	31.3	N/A	763	29	N/A	1215	29.9	N/A
	Depression	Hopkins Symptom Checklist 25 items (HSCL-25)	452	44.1	N/A	763	37.9	N/A	1215	40.2	N/A
	Anxiety	Hopkins Symptom Checklist 25 items (HSCL-25)	452	38.8	N/A	763	27.7	N/A	1215	31.8	N/A
**von Haumeder et al. 2019** [36]	PTSD	PCL-5	43	33.9	18	84	48.8	41	127	46.5	59

*N/A* not available

^a^Weighted prevalence rates

Table 3 summarizes the risk of bias assessments conducted across the 11 included studies. All studies provided a detailed description of the subjects and setting, a standard and reliable measure of mental illness for all participants and appropriate methods for statistical analysis [18, 28–37]. Two studies failed to address low response rates and one study had low response rates for certain subgroups over others [31, 34]. Most studies did not provide an appropriate sampling frame (*n* = 8), utilize random probabilistic sampling (*n* = 9), reach adequate sample size as determined by a power analysis (*n* = 6) or apply valid, diagnostic criteria for mental illness (*n* = 10) [18, 28–37]. The mean score for the risk of bias was 5.72 as two studies failed to score the required 5/9 for inclusion in the meta-analysis [31, 34].

**Table 3:** Risk of bias assessment

Author and Year	Appropriate sample frame	Appropriate sampling of participants	Adequate sample size	Detailed description of subjects and the setting	Data analysis: sufficient coverage of the identified sample	Valid methods for identification of the condition	Standard and reliable measure of the condition for all participants	Appropriate statistical analysis	Adequate response rate or the appropriate management of low response rate	Total score
**Ahmad et al. 2020** [27]	N	N	Y	Y	Y	N	Y	Y	Y	6
**Al Ibraheem et al. 2017** [28]	N	N	N	Y	Y	Y	Y	Y	Y	6
**Chung et al. 2018** [19]	N	N	Y	Y	Y	N	Y	Y	Y	6
**Euteneuer et al. 2018** [29]	N	N	N	Y	Y	N	Y	Y	Y	5
**Georgiadou et al. 2018** [30]	Y	N	N	Y	N	N	Y	Y	N	4
**Javanbakht et al. 2019**	Y	Y	N	Y	Y	N	Y	Y	Y	7
**Lies et al. 2020**	N	N	Y	Y	Y	N	Y	Y	Y	6
**Javanbakht et al. 2019** [31]	N	N	N	Y	Y	N	Y	Y	N	4
**Strømme et al. 2020**	N	N	N	Y	Y	N	Y	Y	Y	5
**Tinghög et al. 2017**	Y	Y	Y	Y	Y	N	Y	Y	Y	8
**Lies et al. 2020** [32]	N	N	Y	Y	Y	N	Y	Y	Y	6
**Mean score**										5.72

Table 4 summarizes the pooled prevalence rates of all three mental disorders by total sample and gender, based on the meta-analysis. Forrest plots representing pooled prevalence rates for all three mental disorders as well for females and males, respectively can be found in the Additional file 1. From the 4873 refugees included in the meta-analysis the total pooled prevalence rate estimate for having either anxiety, depression or PTSD was 33% (CI 95%, 27-40%), 40% for anxiety (CI 95%, 31-50%), 31% for depression (CI 95%, 20-44%) and 31% for PTSD (CI 95%, 22-41%). Heterogeneity for all reported pool prevalence rates were high (I^2^ > 70%). There was no evidence for the presence of publication bias or significant small-study effects (all *p* > 0.05). Subgroup analyses revealed that the combined and individual prevalence rates of all the three mental disorders did not significantly vary by gender. Furthermore, a meta-regression revealed that the total prevalence rates of the common mental disorders reported did not vary significantly by age (*p* = 0.180), duration in host country (*p* = 0.093), host country (*p* = 0.052), educational status (*p* = 0.811) or marital status (*p* = 0.80). The Additional file 1 provides further details regarding the results of the meta-regression.

**Table 4:** Pooled prevalence of mental disorders

	Anxiety depression or PTSD	Anxiety	Depression	PTSD
**Pooled prevalence (95% CI)**	33% (27-40)	40% (31-50)	31% (20-44)	31% (22-41)
Studies (ES)	9 studies (15 ES)	3 studies	5 studies	7 studies
I^2^%	96.86	82.05	97.55	95.26
**Gender**				
Women (95% CI)	34% (27-42)	50 (36-65)	39 (23-57)	33 (20-48)
Studies (ES)	7 studies (13 ES)	3 studies	4 studies	6 studies
I^2^%	97.3%	83.41	96.87	92.99
Men (95% CI)	34% (27-41)	33 (23-43)	26 (16-38)	30 (21-40)
Studies (ES)	8 studies (14 ES)	3 studies	5 studies	6 studies
I^2^%	94.90	74.22	95.72	92.14

*ES* estimates of prevalence rate

## Discussion

We conducted a systematic review and meta-analysis of peer-reviewed publications reporting on the prevalence rates of PTSD, depression and anxiety in adult Syrian refugees resettled in high-income Western countries. Given the humanitarian crisis caused by the ongoing war and violent social upheaval in Syria, it is important to understand the psychiatric comorbidity in this subset of people who currently make up the largest group within the worldwide refugee population. Following our systematic review searches, 11 studies met our inclusion criteria and nine of these papers were deemed appropriate to be included in the meta-analysis following an assessment of publication bias and methodological quality.

Most of the included studies studied resettled Syrian adult refugees in Europe which aligns with resettlement patterns to high-income Western countries of this population [8]. Most of the included studies had either moderate to high methodological quality but were limited by cross-sectional designs, convenience sampling, small sample sizes and self-administered screening questionnaires. Given the small number of studies extracted also broadened the confidence interval of each meta-analysis, the generalizability of our findings must be interpreted with caution. Whilst these factors creates publication bias and reduces methodological quality, it is well established that refugee populations who resettle in foreign countries are hidden and hard to access for research purposes [38]. Resettled refugee communities are often small and there may be concerns regarding the motives of researchers, power imbalances and confidentiality, particularly in the context of mental health where stigma remains high in Arab culture [38, 39].

Although we aimed to limit study variation by focusing on adult Syrian refugees resettled in high-income Western countries, there was still significant heterogeneity in reported prevalence rates. Reported prevalence rates ranged from 9 to 84% for PTSD, 27-53.6% for depression and 13.5-60% for anxiety. Notably, many of these outliers were from the two studies that were ultimately excluded in the meta-analysis due to high publication bias and poor methodological quality. Reported prevalence rates for the studies included in the meta-analysis ranged from 9 to 62.3% for PTSD, 28.7-53.6% for depression and 31.8-53.6% for anxiety. These large variances in reported prevalence rates have similarly been noted in a systematic review looking at Iraqi refugees who have resettled in Western countries where differences in sampling, measurement tools and visa status were noted as potential explanations for these variances [20]. Whilst the meta-regression conducted did not establish a cause for the significant heterogeneity reported, other sources of heterogeneity may have come from differences in participant demographics, employment status, assessment tools used, the nature and severity of exposure to traumatic events and varying lengths since displacement. However, we could not include several of these variables in the meta-regression due to the low number of studies included and the limited reporting on such datapoints.

The pooled prevalence rates of all three mental illnesses reported in this meta-analysis are significantly higher than the general population and are comparable to other meta-analyses conducted within the adult refugee and displaced persons population [8, 12, 40]. Our reported pooled prevalence rates were much lower than the figures estimated by Peconga et al. for depression and PTSD, but not anxiety where only two studies in high-income Western countries were included by the study authors [15]. These discrepancies suggest that the prevalence rates of mental disorders may be higher in Syrian refugees resettled in LMIC. Our non-significant findings associating socio-demographic factors such as age, gender, education levels, marital status and duration in host country with the reported prevalence rates of mental illness also aligns with past meta-analyses conducted within the adult refugee and displaced persons population [8, 12]. Henkelmann and colleagues suggest that the non-significant findings for the influence of duration in host country may indicate that time does not play an important role in the healing or recovery process from post-traumatic events. However, these conclusions must be met with caution given the meta-regressions performed in the present study and in Henkelmann et al.’s meta-analysis were dependent on study averages [12].

Notably, our findings are in contrast with one meta-analysis finding that mental health outcomes were worse in female, older and more educated refugees and IDPs and another reporting a significantly higher prevalence of PTSD in refugee and asylum seeker women [10, 41]. Blackmore and colleagues suggest that the higher prevalence of PTSD in women may be related to a greater risk of exposure to sexual violence, exploitation, trafficking as well as safety concerns, especially ones that are related to child-bearing. Whilst we also reported a higher prevalence rate of all three mental illnesses in female refugees, none were statistically significant. Further research in the Syrian asylum seeker and IDP population would be valuable in elucidating whether gender differences exist in the prevalence of mental illness. Porter & Haslam’s (2005) meta-analysis hypothesized that younger refugees are more resilient and less prone to mental illnesses whilst more educated refugees are subjected to a greater loss of status and have a higher prevalence of mental illness [10]. However, comparisons to this paper are limited by the authors’ inclusion of IDPs, asylum seekers and stateless persons in their study [10]. To the best of our knowledge, no meta-analysis has to date reported on the associations of marital status with mental illness in the refugee population.

Our study has many strengths. To the best of our knowledge, this is the first study to systematically review and pool prevalence rates for mental illness in the adult Syrian refugee population who have resettled exclusively in high-income Western countries. We used a comprehensive list of search terms, covering many ethnic minority groups in Syria and conducted these searches across seven electronic databases. We used rigorous and objective measures to assess for eligibility and evaluate for publication bias. Lastly, we conducted sensitivity analyses, tested for heterogeneity and were able to moderate the effect of demographic factors and conduct sub-group analyses to elucidate for confounding effects.

### Limitations

However, a few limitations in this study must be noted. We excluded unpublished studies, studies that were not written in English and studies where a proportion of the sample were not Syrian refugees. This will have limited the number of studies included in the review. Most studies in the current review used non-probability sampling methods which reduces the overall generalizability to the resettled adult Syrian refugee population due to the introduction of selection bias. Moreover, we observed high levels of heterogeneity between the included studies which were not explained for by the meta-regression conducted. Though certain variables were unable to be included in the meta-regression (e.g., types of traumatic events encountered), high heterogeneity is often a central feature in meta-analyses of prevalence rates [42]. Furthermore, only one study used a formal diagnostic interview to assess for mental illness. Thus, our pooled prevalence rates are likely to be overestimated. However, it must be noted that the resettled refugee population is small and often hard to reach as well as access for research purposes [38]. Finally, although every effort was put into obtaining further information from corresponding authors, in cases where there was no response, we were also limited by the amount of data made available in the published articles and the varying sociodemographic variables collected by each research group.

## Conclusion

Our study demonstrates the high prevalence of PTSD, depression and anxiety in adult Syrian refugees who resettle in high-income Western countries. These findings are of clinical significance, given the high numbers of Syrian civilians who have sought refugee status in high-income Western countries and more importantly, the large population of Syrian asylum seekers and IDPs who have yet to permanently resettle. In response to these growing public health concerns, large scale mental health programs and interventions catered to Syrian refugees resettled in high-income Western countries have been trialled but with mixed findings thus far [43, 44]. Thus, we believe that more research should be conducted to better understand the morbidity, pre- and post-displacement risk factors of developing mental illness in this vulnerable population, given the violent and psychological traumas afflicted by the war in Syria. Gaining a greater understanding of these factors will ultimately enable clinicians to better tailor culturally sensitive prevention and treatment programs earlier on in the resettlement process. Moreover, future epidemiological research in this population should ideally use population-based sampling and diagnostic tools, validated in the language that the tool is administered in.

## Supplementary Information

**Additional file 1: Appendix** **A** – PRISMA 2020 Checklist. **Appendix B** - Search strings. **Appendix C** – Forrest plots. **Appendix D** – Meta-regression analyses.

## Data Availability

Data and materials used during the present study are available upon reasonable request from the corresponding author.

## Abbreviations

DSM-5: Diagnostic and Statistical Manual of Mental Disorders, 5th Edition: DSM-5
IDPs: Internally displaced persons
JBI: Joanna Briggs Institute
LMIC: Low/middle income countries
PRISMA: Preferred Reporting Items for Systematic Reviews
PTS: Political Terror Scale
PTSD: Posttraumatic Stress Disorder
UNHCR: United Nations High Commissioner for Refugees
